# Silk fibroin–collagen hydrogel loaded with IGF1-CESCs attenuates intervertebral disk degeneration by accelerating annulus fibrosus healing in rats

**DOI:** 10.3389/fphar.2025.1552174

**Published:** 2025-03-27

**Authors:** Zhiqiang Tian, Zigang Shen, Hong Chen, Ping Zhao

**Affiliations:** ^1^ Biological Science Research Center, Integrative Science Center of Germplasm Creation in Western China (CHONGQING) Science City, Southwest University, Chongqing, China; ^2^ Sericulture Science and Technology Research Institute of Chongqing, Chongqing, China; ^3^ Department of Orthopedics, 903 Hospital of Joint Logistic Support Force of The People’s Liberation Army, Hangzhou, China

**Keywords:** annulus fibrosus, wound treatment, IGF1, exosome, intervertebral disc

## Abstract

**Introduction:** The self-healing capacity of a damaged annulus fibrosus (AF) leads to intervertebral disk (IVD) degeneration. AF wound treatment is challenging. The combination of biomaterials and stem cell-derived exosomes is a promising wound treatment strategy with significant clinical value.

**Methods:** We isolate primary nucleus pulposus cells (NPCs) and primary annulus fibrosus cells (AFCs) from rats as the target cells of rat insulin-like growth factor 1(IGF1), and verify the proliferation and migration; constructed cartilage endplate stem cells (CESCs) engineered cells that release exosomes containing high concentrations of IGF1by lentiviral infection, and used the IGF1-CESCs combined with combined silk fibroin (SF) and a collagen-mixed hydrogel for the treatment of AF wounds in rat.

**Results:** We found that both IGF1 and IGF1-rich exosomes (IGF1 Exo) promoted the proliferation and migration of AFCs. SF and collagen mixed hydrogels have excellent compressive mechanical properties and are suitable for use in IVD therapy. After the IGF1-CESCs@SF-collagen hydrogel was filled in the damaged area of the AF in rat, the wound healing was accelerated, nucleus pulposus overflow was inhibited, the IVD height was maintained, and degeneration was reduced.

**Discussion:** The IGF1-CESCs@SF-collagen hydrogel can efficiently treat AF wounds and inhibit degeneration of IVD, and has potential for clinical treatment.

## 1 Introduction

Lower back pain (LBP) seriously affects the life of patients, leads to a large loss of labor, and increases the medical burden on the society (Freemont, 2009). The annulus fibrosus (AF) and nucleus pulposus (NP) are important tissues that support the height of the intervertebral disk (IVD) and enable the spine to bend. Long-term failure to heal after damage induces IVD degeneration, eventually resulting in LBP. Particularly, rapid AF repair after damage is crucial for maintaining IVD homeostasis (Sloan et al., 2020). The timely supply of trophic factors is essential for the wound treatment of the damaged IVD. Insulin-like growth factor-1 (IGF1) is a cytokine closely related to bone growth (Zhang et al., 2020) and can regulate angiogenesis and bone regeneration. IGF1 inhibits the apoptosis of nucleus pulposus cells (NPCs) and activates the Akt pathway to release the extracellular matrix (ECM) such as aggrecan and collagen II (Xiao et al., 2021; Chen et al., 2020); it is also considered crucial in wound healing of the damaged AF (Elmasry et al., 2016).

Stem cells have been widely studied in regenerative medicine, but their senescence, tumorigenicity, and low efficiency in directed differentiation limit their clinical applications. Exosomes derived from stem cells not only retain the excellent characteristics of stem cells but also avoid the limitations of stem cells, and they have a wider range of clinical application prospects (Tan et al., 2024). Combinations of stem cells and exosomes are widely used for wound treatment (Rani and Ritter, 2016) and have attracted considerable attention for their use in repair after the damage of IVD (Luo et al., 2022). In contrast to exogenous stem cells (mesenchymal stem cells, skeletal stem cells, and adipose-derived stem cells), cartilage endplate stem cells (CESCs) are derived from the cartilage endplate (CEP) tissue of the IVD itself and have considerable potential for bone repair, IVD wound treatment, and other applications. We found that CESCs could release exosomes to induce NPC differentiation, thus repairing the NP (Luo et al., 2021a). We induced CESCs to stably express *Sphk2* and release Sphk2 protein-rich exosomes and then combined them with the costal chondrocyte extracellular matrix and collagen hydrogel to form an injectable Sphk2-CESC-exosome hydrogel, which effectively attenuated IVD degeneration (Luo et al., 2022). Although loose collagen hydrogels are beneficial for the growth of CESCs, because of their low mechanical pressure-bearing capacity, they cannot resist pressure from the spine, which limits their potential for clinical application. Silk fibroin (SF) has advantages such as good biocompatibility, adjustable degradability, and low toxicity, and it has been widely used as a dressing and pharmaceutical ingredient in wound treatment research (Farokhi et al., 2018). SF is also considered the best natural biomaterial for treating and reconstructing AF wounds (Wang et al., 2022; Bhattacharjee et al., 2012). In recent years, biomaterials such as collagen I/II, glycosaminoglycans, and pentosan polysulfate combined with stem cells have been widely favored for treating IVD. SF is highly favored in the medical field owing to its low immunogenicity and good biocompatibility. However, relatively few studies have examined the combination of silk fibroin and CESCs as biomaterials for IVD diseases.

In this study, we re-examined the relationship between IGF1 in AF wound treatment and IVD degeneration and verified its effects on the proliferation and migration of NPCs and annulus fibrosus cells. We constructed CESCs that stably transcribed the rat *igf1* gene, released exosomes rich in rIGF1 protein, and transformed them into a rIGF1-CESCs@SF-collagen hydrogel. This injectable hydrogel exhibited excellent mechanical properties and a strong ability to repair damaged AF, which can thus be an effective treatment strategy to attenuate IVD degeneration.

## 2 Materials and methods

### 2.1 Reagents and antibodies

Antibodies to glyceraldehyde 3-phosphate dehydrogenase (GAPDH) (cat. 60004-1-Ig), Cytl1, HSP70, Alix, and cleaved caspase-3 were purchased from Proteintech (Wuhan, China). Antibodies to IGF1 and KRT19 were purchased from BOSTER. DAPI nucleic acid dye (cat. C1006) and crystal violet staining solution (cat. C0121) were purchased from Beyotime Biotechnology (Shanghai China). Anti-TSG101 was obtained from ABclonal (cat. no. A1692, Wuhan, China). Goat anti-rabbit IgG  (cat. ab150080, Alexa Fluor^®^ 594) and Goat anti-rabbit IgG (cat. ab150077, Alexa Fluor^®^ 488) were obtained from Abcam (Cambridge, MA, USA). The EdU Cell Proliferation Kit with Alexa Fluor 594 (cat. C0078S) was obtained from Beyotime Biotechnology (Shanghai, China). Type 2 (cat. A004174-0001) and type 4 collagenase (cat. A004186-0100) were purchased from Sangon Biotech (Shanghai, China).

### 2.2 Patient tissues and histological analysis

Clinical specimens were obtained from 15 patients undergoing surgery at the Department of Orthopedics of 903 Hospital of Joint Logistic Support Force of The People’s Liberation Army. The severity assessment of IVD degeneration follows the modified Pfirrmann magnetic resonance imaging (MRI) method. Those with grades 1 and 2 were classified as having mild degeneration, whereas those with grades above 2 were considered as having severe degeneration. They were divided into several parts. Some of the tissues were frozen in liquid nitrogen for immunofluorescence and RT-qPCR analysis; others were fixed in 4% formalin buffer, decalcified with 10% ethylenediaminetetraacetic acid, embedded in paraffin after dehydration, and then used to perform tissue sectioning. The tissue slices were soaked in citrate buffer (0.01 M) at 110°C for 15 min, in 3% hydrogen peroxide for 15 min, and in 5% bovine serum albumin (BSA) for 40 min. The tissue sections were incubated with the IGF1 primary antibody (1:200) for 6 h at 4°C, and they were incubated with the secondary antibody (horseradish peroxidase-conjugated) for 1 h at 37°C. They were detected via 3,3′diaminobenzidine tetrahydrochloride (DAB) staining. This study was approved by the Medical Ethics Committee of 903 Hospital of Joint Logistic Support Force of The People’s Liberation Army (NO. 20241229/49/01/003), and all included patients provided informed consent.

### 2.3 Immunofluorescence

Immunofluorescence detection follows the method we previously used (Luo et al., 2022), which is briefly described as follows: the tissue sections were incubated in 10% hydrogen peroxide/formaldehyde buffer for 35 min at 37°C. They were then treated with 0.2% Triton for 5 min, blocked with 5% (w/v) BSA, and incubated with the primary antibody (IGF1, 1:00; cleaved caspase 3, 1:50) for 6 h. They were then incubated with a fluorescent secondary antibody and stained with 4′,6-diamidino-2-phenylindole. Images were captured using a fluorescence microscope (Olympus, Tokyo, Japan).

### 2.4 Animal experiments

Twenty-four 1-month-old Sprague–Dawley rats were purchased from the Experimental Animal Center of the Army Military Medical University and divided into four experimental groups: a healthy control group (n = 6), acupuncture group (n = 6), rIGF1-CESCs@SF-collagen hydrogel group (n = 6), and CESCs@SF-collagen hydrogel treatment group (n = 6). The injury was induced, as described by Han et al. (2008). The rats were anesthetized with Delivector™ Avertin (10–20 μL/g). The AFs were damaged using a 21-gage needle. The needle was inserted into the AF, but no damage was caused to the NP tissue. After the induction of the injury was completed, in the rIGF1-CESCs@SF-collagen hydrogel group and the CESCs@SF-collagen hydrogel treatment group, 10 μL of rIGF1-CESCs@SF-collagen hydrogel and CESCs@SF-collagen hydrogel were injected into the damaged sites, respectively. The rats were sacrificed at weeks 1 and 6, respectively. IVD tissues were isolated for immunofluorescence and hematoxylin and eosin (HE) staining. Images were captured and analyzed (Olympus, Tokyo, Japan). The handling of rats complies with the requirements of the Guidelines for the Care and Use of Laboratory Animals of the National Institutes of Health, and the approval number from the Animal Ethics Committee of Army Medical University is AMUWEC20230455.

### 2.5 Separation of AFCs and NPCs

The cells were derived from the IVD of 2-week-old male rats. First, the isolated rat tail IVD was washed with 0.1 M sterile phosphate-buffered saline (PBS). NP and AF tissues were peeled off from the IVD tissue and cut into fragments by mechanical cutting. The tissues were digested with collagenase II (0.2%) for 3.5 h at 37°C. A filter was used to remove undigested clumps, and the clumps were washed with PBS. They were centrifuged at 1,000 rcf for 10 min. The isolated cells were cultured with the medium containing 10% fetal bovine serum and 1% penicillin–streptomycin in 5% CO_2_ at 37°C. The medium was changed every 2 days. Cells were passed to the second generation for subsequent experiments. CESCs come from our laboratory’s previous preservation (Luo et al., 2022).

### 2.6 Gene Ontology enrichment analysis

The analysis data are obtained from our previous research (Luo et al., 2021a). The overview is as follows: exosomes are extracted from CESCs cultured *in vitro*, and proteins are extracted from exosomes for protein mass spectrometry analysis. The top 200 proteins with the most abundant expression are selected, Gene Ontology (GO) enrichment analysis is performed by WeChat online (https://www.bioinformatics.com.cn/?keywords=pathway), and GO enrichment analysis graphs are created using GraphPad prism 10.3.

### 2.7 Wound healing and transwell assay

AFCs or NPCs (5 × 10^5^/well) were inoculated into a 6-well plate. When the confluence reached 80%, a pipette tip was used to create a straight scratch. They were washed with PBS containing 10% fetal bovine serum. Scratches were photographed under an optical microscope at 0, 24, and 48 h. For the cell migration analysis, an appropriate amount of complete medium was added to a 24-well plate, the transwell chamber was placed in the 24-well plate, and the cell suspension was added. After 24 h, the chamber was removed and stained with crystal violet to determine the number of cells that passed through the membrane.

### 2.8 Lentivirus transfection

Lentiviruses to overexpress *rIGF1* were obtained from Honglian (Chongqing, China). When the CESCs reached 60% confluence, the *rIGF1*-lentivirus was added to the culturing cell, referring to the method (Luo et al., 2023) we previously used for screening positive cells. We analyzed the transfection efficiency by RT-qPCR and Western blotting.

### 2.9 Quantitative reverse transcription polymerase chain reaction (RT-qPCR) analysis

Total RNA was extracted from tissue or cells using TRIzol and was reverse-transcribed into cDNA using an RT kit (Takara, Japan). Three independent qPCR detections were performed using a Bio-Rad CFX96 qPCR machine (Bio-Rad Laboratories, Hercules, CA, USA), with GAPDH as the control to assess the gene expression. The primer sequence is as follows: *h(human)Igf1-F*: 5′-TGA AGG GAG GTG GTG GGT AT-3′, R:5′-CTC TGA ATC TTG GCT GCT GG-3’; *hGapdh*-F: 5′-TGG AAG ATG GTG ATG GGA TT-3′, R:5′-CTC TGA TTT GGT CGT ATT GGG-3’; *r(rat)Igf1*-F: 5′-TAA GAA AGG GCA GGG CTA AT-3′, R:5′-TTT ATA GGT GGT TGA TGA ATG G-3’; *rCytl1-F*: 5′-TGT GAC AGG GTA CTG CCC AC-3′, R:5′-TCC CAT GAT GCA ATC GTT GA-3’; *rCol1α1*-F: 5′-GAT TGG GAT GGA GGG AGT TTA-3′, R:5′-TAC AGC ACG CTT GTG GAT GG-3’; *rKrt19*-F: 5′-AAG TCG CAC TGG TAG CAA GG-3′, R:5′-ATC AAG TCG AGG CTG GAG CA-3’; *rSox9*-F: 5′-CAA CAG ATG ACC ATA CCC TTT-3′, R:5′-TCA CCT GTA CCT CCC TGA ATA-3’; *rGapdh*-F: 5′-CGC CAG TAG ACT CCA CGA CAT-3′, R:5′-CGG CAA GTT CAA CGG CAC AG-3’.

### 2.10 Western blotting

Tissue or collected cells were lysed in RIPA buffer containing phenylmethylsulfonyl fluoride and extracted. The protein concentration of the supernatant was determined using a spectrophotometer (Beckman, Fullerton, CA, USA). Proteins were separated by polyacrylamide gel electrophoresis, and they were transferred to a polyvinylidene fluoride (PVDF) membrane by electroblotting. The PVDF membrane was placed in a closed solution containing 5% (w/v) skim milk for 1 h, incubated with antibodies (1:1000–2000) for 6 h at 4°C, and then washed thrice with PBS and incubated with secondary antibodies (horseradish peroxidase-conjugated) for 1 h at 37°C. The PVDF membrane was soaked in an ECL working solution (Millipore, MO, USA) for color development. Finally, the proteins were detected, and images were captured using the chemiluminescence system (Bio-Rad Laboratories).

### 2.11 Preparation of the SF–collagen hydrogel

Rats were anesthetized and sacrificed by cervical dislocation. The skin on the tail was incised, and the tail tendons were removed and washed with normal saline. The tail tendons were chopped and placed in 150-mL acetic acid solution (0.5%). They were then placed on a shaker and dissolved at 4°C for 48 h. Next, the mixture was centrifuged at 12,000 rcf for 15 min. The supernatant was aspirated and added to a 10% NaCl solution to precipitate the collagen. An appropriate amount of hydrochloric acid (0.2 mM) was added to dissolve the supernatant, and the collagen concentration was adjusted to 10 mg/mL. The collagen solution was stored at 4°C for later use.

Two grams of silkworm cocoons was weighed, placed into 1 L of a 0.5%–1% Na_2_CO_3_ solution, and boiled in water for 60 min to remove the sericin. The silk fibroin fibers were washed three times with 100 mL of double-distilled water, placed in an oven, and baked at 60°C for 30–50 min. A ternary solution was prepared by mixing 30.43 mL dd H_2_O, 19.57 mL ethanol, and 24 g calcium chloride. The silk fibroin was placed in the ternary solution and dissolved at 60°C for 1–2 h. Centrifugation was performed at 4,000 rcf for 15 min, and the supernatant with the silk fibroin was collected. The silk fibroin solution was then transferred into a dialysis bag with a molecular weight cutoff of 8,000–14,000 D for 48 h. Centrifugation was performed at 4,000 rcf for 10 min, and the supernatant was collected to detect the concentration of the SF solution. The SF solution was concentrated with 15% polyvinyl alcohol 20000 to obtain a protein concentration of 8%–15%. Finally, collagen and SF solution were mixed at a ratio of 4:1 (the higher concentration of SF is not conducive to cell survival) to prepare the hydrogel.

### 2.12 Transmission electron microscope (TEM)

Extracellular vesicles (Luo et al., 2022) were collected and fixed with glutaraldehyde (3%) for 10 h. Osmium acid (1%) was fixed for 1.5 h. Gradient dehydration, embedding, sectioning (70–90 nm), and staining were performed. The sample was observed by using a TEM (Philips, Amsterdam, Netherlands).

### 2.13 Scanning electron microscope (SEM)

SF and collagen were mixed into a hydrogel, cooled at 20°C for 30 min, installed on a fixed frame and cooled at −70°C for 2 h, and then placed in a freeze dryer for 12 h; after gold plating and slicing for detection, images were captured using the SU3500 SEM (Hitachi, Tokyo, Japan).

### 2.14 Statistical analysis

The results were presented as the mean ± standard deviation. GraphPad Prism 7.0 (GraphPad Software, Inc., CA, United States) was used for statistical analyses. One-way analysis of variance or Student's t-test was used to compare the means between groups. A *p-*value of less than 0.05 was statistically significant.

## 3 Results

### 3.1 IGF1 expression in human and rat IVDs

**FIGURE 1:**
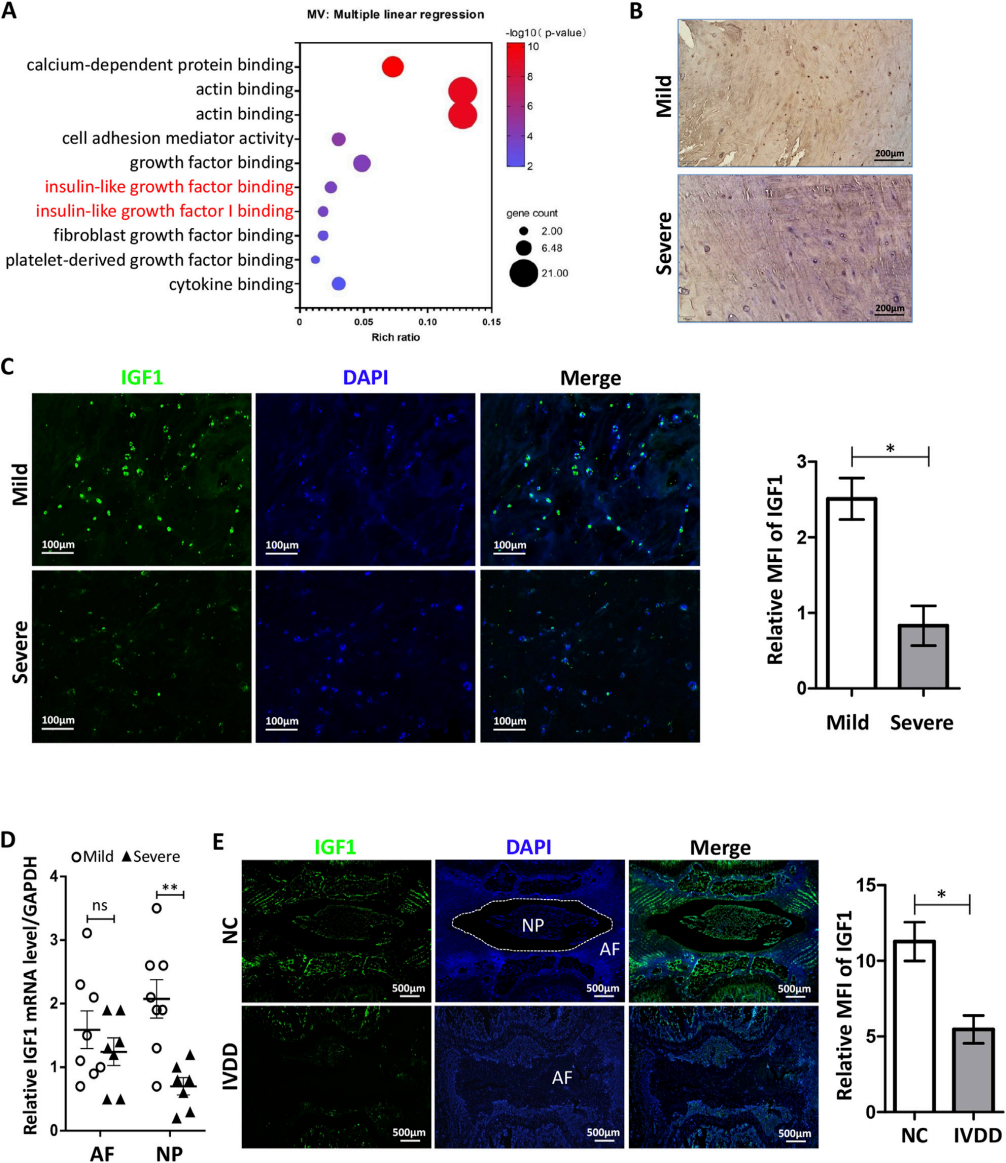
Expression of IGF1 in human and rat IVDs. **(A)** Enrichment analysis of CESCs-Exo carrier proteins in healthy rats using GO enrichment analysis (Luo et al., 2021a). **(B)** Immunohistochemistry was performed to detect IGF1 (green) expression in degenerated IVD tissues in patients. **(C)** Expression of IGF1 in patients degenerative IVD tissues was detected by immunofluorescence. **(D)** RT-qPCR was used to detect the expression of *Igf1* in the NP and AF in patients degenerative IVD tissues (mild, n = 8; severe, n = 7). **(E)** Punctures induced degeneration in the rat tail IVD, and after 6 weeks, the protein expression and tissue localization of IGF1 (green) in the tail IVD of healthy and degenerated rat tissues were detected by tissue immunofluorescence. Data in **(C–E)** are presented as the mean ± SD,*p < 0.05, **p < 0.01, ns: not significant by two-tailed Student’s t-test.

We isolated CESC-derived exosomes from rats in previous research (Luo et al., 2021a). Through proteomic mass spectrometry detection and GO enrichment analysis, we found that the degree of activation of proteins related to the IGF pathway was abnormally high (Figure 1A). IGF is an important regulatory factor for bone growth and the main stimulatory factor for ECM synthesis. We speculate that CESCs release IGF-containing exosomes to maintain IVD homeostasis.

We collected tissues from patients who underwent IVD removal and performed pathological examinations. Immunohistochemical analysis showed that IGF1 in IVD tissues with mild degeneration was significantly higher than that in tissues severe degeneration (Figure 1B). The immunofluorescence and qRT-PCR results were similar to those of immunohistochemistry (Figures 1C,D), and there are significant differences in the number of IGF1-positive cells between patient tissues with mild and severe degeneration (Figure C). We induced degeneration of the rat tail IVD using a puncture method. At the 6th week, we used tissue immunofluorescence to detect the expression and localization of IGF1 in the rat tail IVD and found that it mainly existed in the CEP and NP regions of healthy IVD. The healthy IVD group had a higher expression of IGF1 protein than the degenerated IVD group (Figure 1E). These results indicate that IGF1 is crucial for maintaining the homeostasis of IVD.

### 3.2 Isolation and identification of rat AFCs and NPCs

**FIGURE 2:**
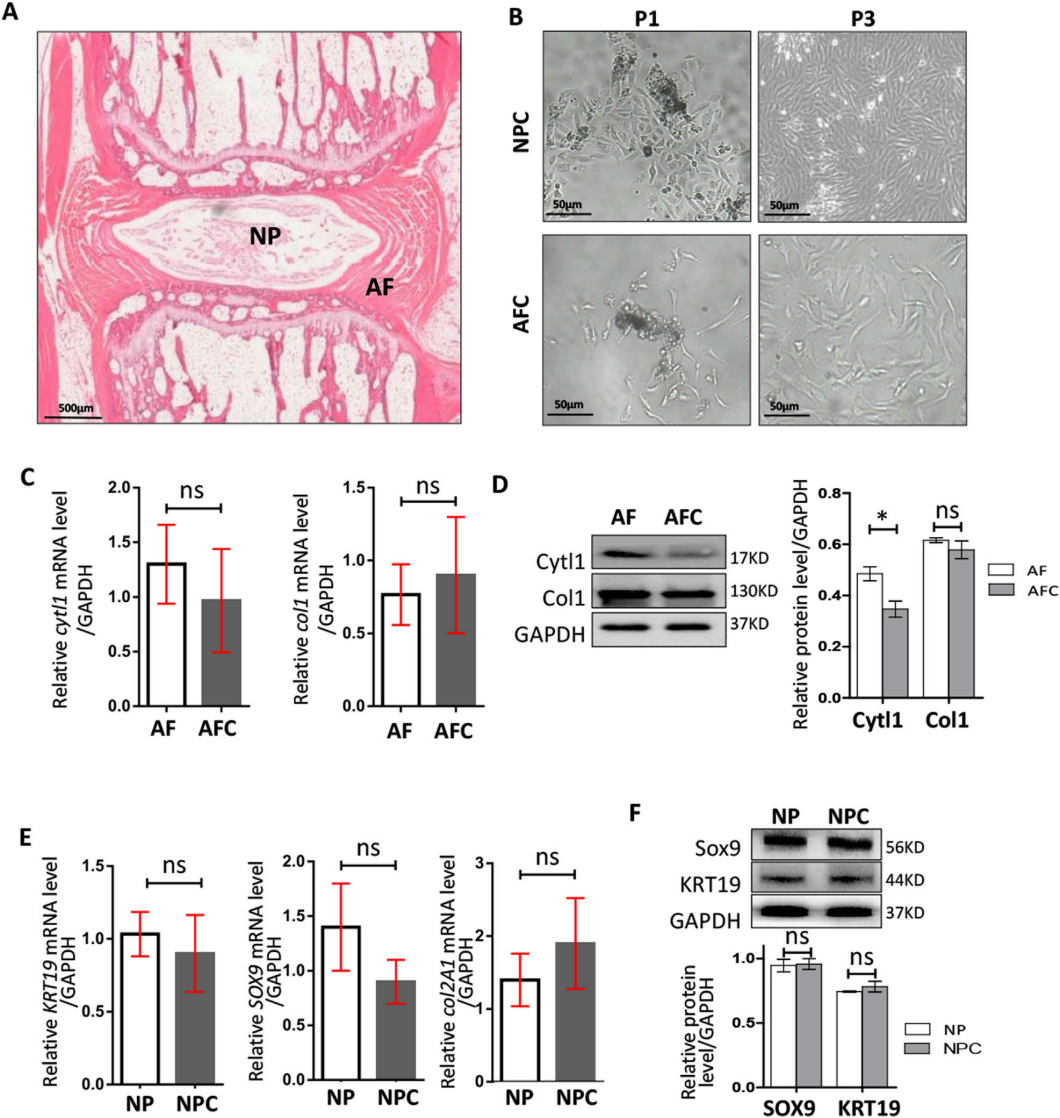
Isolation and identification of rat AFCs and NPCs. **(A)** Morphological map of rat tail IVD using hematoxylin–eosin staining, AF: annulus fibrosus, NP: nucleus pulposus. **(B)** Annulus fibrosus cells (AFCs) and nucleus pulposus cells (NPCs) cultured after tissue dissociation and collagenase digestion, P1: the first generation, P3: the third generation. **(C)** Expressions of *cytl1* and *col1* genes in AF tissue and AFCs were detected by RT-qPCR. **(D)** Western blotting was used to detect the differences in the expressions of Cytl1 and Col1 in AF tissues and AFCs. **(E)** Expression levels of *Sox9*, *krt19*, *and col2a1* genes in the NP and NPCs were detected by RT-qPCR. **(F)** Western blotting was used to detect the differences in the expression levels of Sox9 and KRT19 in the NP and NPCs. Data are presented as the mean ± SD in **(D, F)**, *p < 0.05, ns: not significant by two-tailed Student’s t-test.

To verify the influence of IGF1 on the physiological activities of these cells within the IVD, we isolated NP and AF tissues from rat tail IVD (Figure 2A). The tissues were digested with collagenase to obtain single NPCs and AFCs and then cultured for *in vitro* cell experiments (Figure 2B). We identified differences in the expression of AF-specific markers (*cytl1* and *col1*) (Fernandes et al., 2020; van den Akker et al., 2016) in rat AF tissues and AFCs using RT-qPCR and Western blotting (Figures 2C,D). The expression of NP-specific markers (*krt19* (Rutges et al., 2010), *sox9*, and *col2a1*) in NP tissues and NPCs was determined in a similar manner (Figures 2E,F). These results showed that the characteristics of AFCs and NPCs isolated *in vitro* were highly similar to those of AF and NP tissues, respectively. The AFCs and NPCs that were separated can represent AF and NP tissues, respectively, for *in vitro* experiments.

### 3.3 Effects of IGF1 on the proliferation and migration of AFCs and NPCs

IGF1 contributes to osteocyte proliferation and is involved in the homeostasis of bone tissues (Xian et al., 2012). IGF1 must bind to the IGF receptor (IGFR) of target cells to activate intracellular signaling pathways and perform its functions.

**FIGURE 3:**
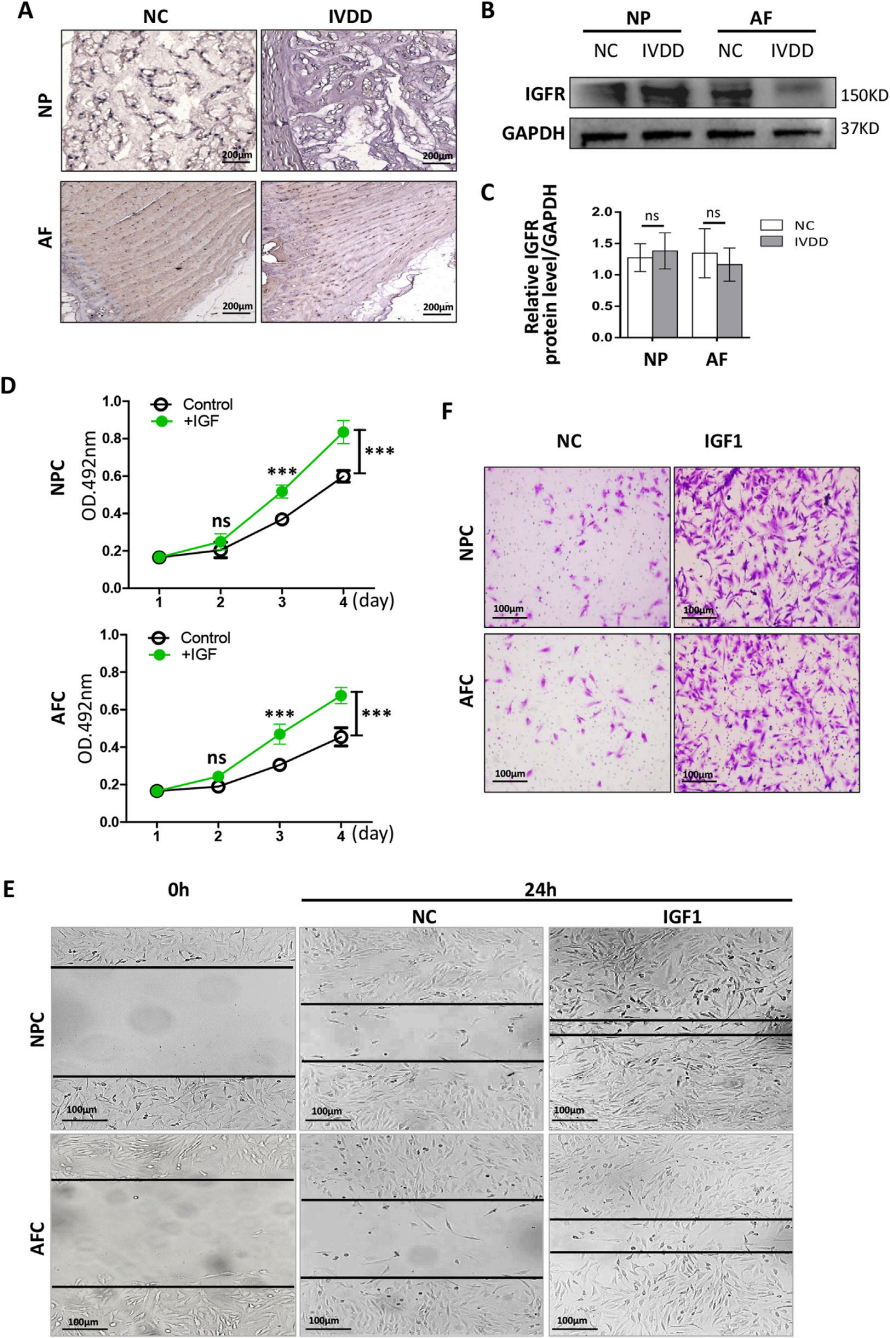
Effects of IGF1 on the proliferation and migration of NPCs and AFCs. **(A)** Immunohistochemical detection of IGFR expression in rat IVD tissue by staining with 3′- diaminobenzidine (NP, healthy; IVDD, degeneration). **(B)** Western blotting was used to detect the differences in the expression levels of IGFR in the AF/NP tissue between NP and IVDD. **(C)** Grayscale analysis of relative expression levels of IGFR protein. **(D)** Recombinant rat IGF1 was added to the AFC and NFC culture medium, and the cells were cultured *in vitro* for 4 days; the cell proliferation was detected by MTT. **(E)** Wound healing and transwell assay **(F)** were used to detect the migration of recombinant rIGF1 on AFCs and NPCs (crystal violet staining solution for cell nuclei). Data are presented as mean ± SD in **(C, D)**, ***p < 0.001, ns: not significant by two-tailed Student’s t-test.

To determine the influence of IGF1 on the proliferation of AFCs and NPCs, we measured the expression of IGFR in rat AF and NP tissues. Immunohistochemistry was performed to detect IGFR in healthy rat tail IVD tissues and those with degeneration. We found that IGFR was expressed in both AF and NP tissues, and its expression showed no significant differences between healthy and degenerated tissues (Figure 3A). Western blotting showed that IGFR expression in AF tissues remained relatively stable, with no differences in IGFR expression between healthy and degenerated AF tissues (Figures 3B,C), aligning with results from previous studies (Le Maitre et al., 2005). There was also no significant difference in the expression of IGFR between healthy NP and degenerated NP (Figures 3B, C).

Recombinant rat IGF1 (rIGF1) (100 ng/mL) was added to the culture media of AFCs and NPCs, and the cells were continuously cultured for 4 days. Subsequently, the MTT assay was used to detect cell proliferation. The rIGF1 effectively promoted the proliferation of AFCs and NPCs (Figure 3D). Wound healing and transwell assays confirmed that IGF1 promoted the migration of AFCs and NPCs (Figures 3E, F). These results suggest that IGF1 can regulate physiological IVD homeostasis by exerting an influence on AF and NP.

### 3.4 Effects of rIGF1-CESC exosomes on NPCs

**FIGURE 4:**
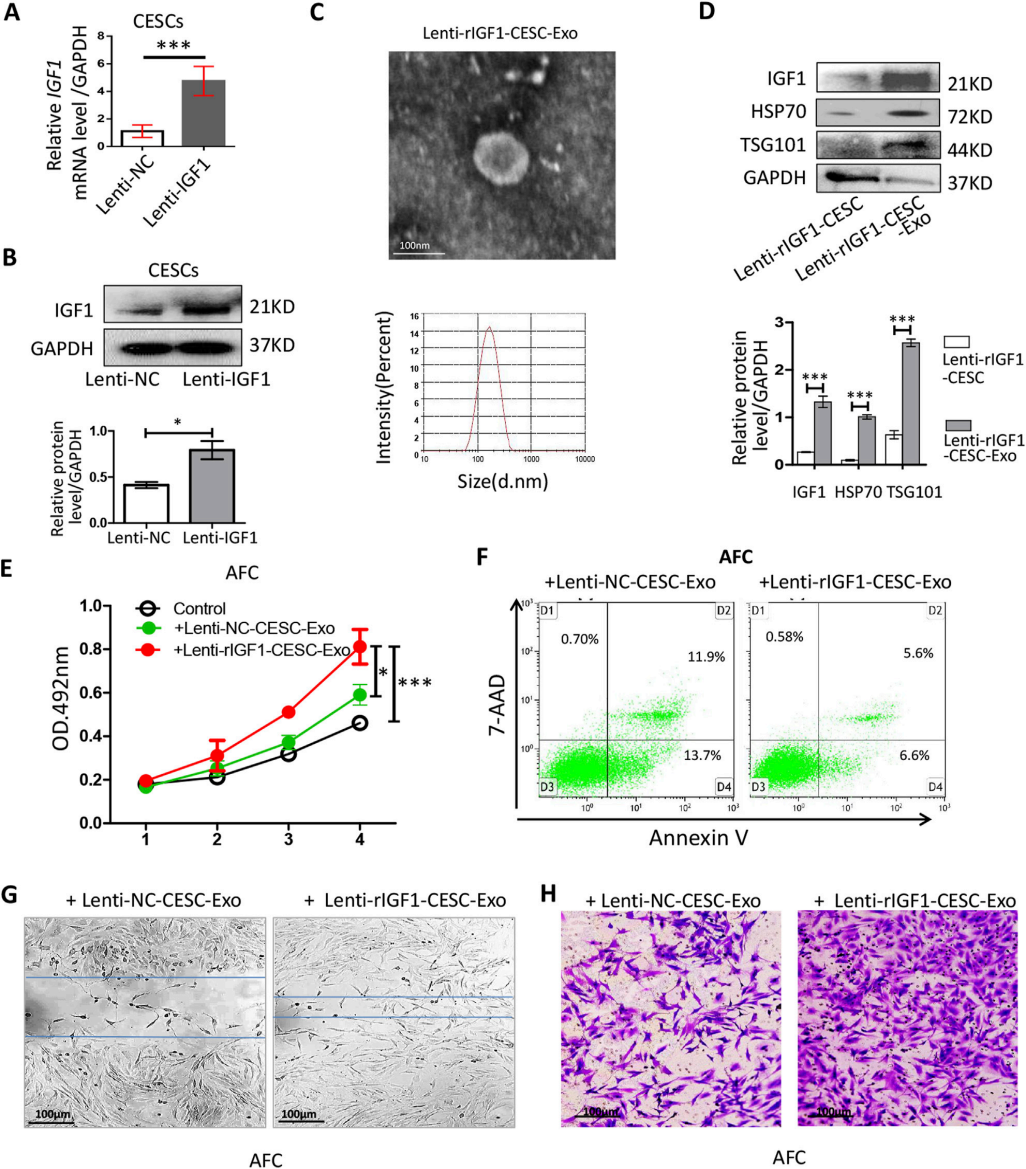
Identification of the activity of lent-*rIGF1*-CESC exosomes on AFCs. **(A)** RT-qPCR was used to detect the level of *rIgf1* mRNA in CESCs transfected with the lentiviral *rIgf1* gene or empty vector (NC). **(B)** Western blotting was used to detect the differences in the expression of IGF1 protein. **(C)** Morphology of lent-*rIGF1*-CESCs-Exo was observed by TEM, and particle size was detected through a particle size analyzer. **(D)** Western blotting was used to detect the expressions of lent-*rIGF1*-CESCs-Exo-specific markers (HSP70 and TSG101) and rIGF1 protein. **(E)** Lent-I *rIGF1*-CESCs-Exo was added to the culture medium of AFCs, which were cultured *in vitro* for 4 days, and the cell proliferation was detected by MTT. **(F)** The AnnexinV/7-AAD kit was used to stain the cultured AFCs, and flow cytometry was used to detect apoptosis. **(G)** The migration of lent-*rIGF1*-CESCs-Exo to AFC cells was detected by wound healing and **(H)** transwell assays (crystal violet staining solution for cell nuclei). Data are presented as the mean ± SD in **(B, D)** by two-tailed Student’s t-test or in **(E)** by one-way ANOVA. *p < 0.05, ***p < 0.001.

We aimed to develop a treatment strategy that could allow rapid healing after AF damage. Increasing the concentration of IGF1 in exosomes enhanced their ability to activate AFCs. We transfected the rat *IGF1* gene into CESCs to construct CESCs-exosomes with a high expression of rIGF1 (IGF1-CESCs-Exo). The *rigf1* gene was transfected into CESCs using a lentivirus. Identification by qRT-PCR and Western blotting showed that lenti-rIGF1-CESCs expressed higher levels of *rigf1* mRNA (Figure 4A) and IGF1 protein (Figure 4B) than the controls. The exosomes were isolated, and their particle size was determined using TEM and particle size analyzer (Figure 4C). The expressions of exosome-specific markers and IGF1 (Figure 4D) were detected by Western blotting, and the results showed that rIGF1-CESCs-Exo was rich in rIGF1 protein.

Exosomes were extracted and added to the AFC culture medium. The MTT assay was used to detect cell proliferation, and rIGF1-CESCs-Exo could increase the growth rate of AFCs (Figure 4E). Flow cytometry was used to detect the apoptosis rate, and the results showed that the ability of rIGF1-CESCs-Exo to inhibit the apoptosis of AFCs was slightly improved compared with that of the control group (Figure 4F). Moreover, wound healing and transwell assays confirmed that rIGF1-CESCs-Exo significantly enhanced the migration ability of AFCs (Figures 4G,H).

These results indicate that promoting the activation and proliferation of AFCs by releasing rIGF1-rich exosomes from rIGF1-CESCs is a potential strategy for repairing damaged AF.

### 3.5 Preparation and identification of SF hydrogel biomaterials

To obtain exosomes of rIGF1-CESC engineered cells, we further prepared materials that could carry cells within the IVD to enable them to proliferate stably and continuously release exosomes containing the IGF1 protein, thereby enhancing the potential for wound treatment.

**FIGURE 5:**
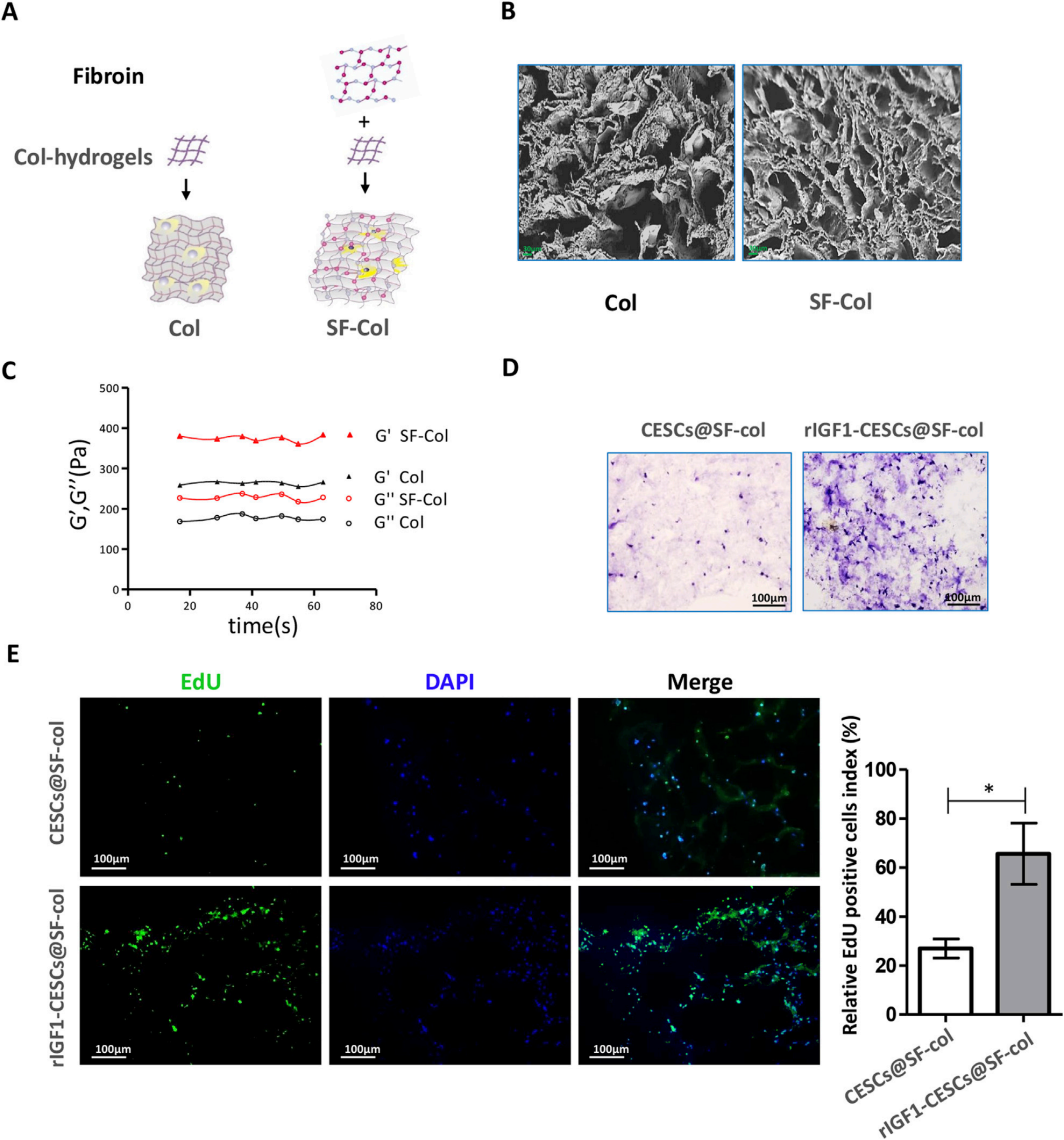
SF hydrogel is beneficial for the proliferation of CESCs. **(A)** SF–collagen and collagen hydrogel preparation diagram. **(B)** SEM was used to observe the SF–collagen hydrogel (silk fibroin: 2.5 mg/mL, collagen: 10 mg/mL) and collagen hydrogel (collagen: 10 mg/mL). **(C)** Analysis of storage modulus and loss modulus of SF–collagen hydrogel and collagen hydrogel. **(D)** Hematoxylin/eosin and EdU staining **(E)** to detect the growth of CESCs with or without IGF1 expression in the SF–collagen hydrogel. CESCs are encapsulated in SF–collagen hydrogels and cultured in the medium containing 10% fetal bovine serum for 72 h. Data are presented as the mean ± SD in **(E)**, *p < 0.05 by two-tailed Student’s t-test.

SF exhibits excellent biocompatibility and is used to treat damaged AF [10]. The SF solution was combined with a collagen hydrogel to form an SF–collagen hydrogel (with a silk fibroin concentration of 2.5 mg/mL and a collagen concentration of 10 mg/mL) (Figure 5A). SEM revealed that the pore size of the SF–collagen hydrogel was slightly smaller than that of the pure collagen hydrogel (Figure 5B). Moreover, both the storage modulus (G′) and the loss modulus (G″) of the SF–collagen hydrogel were higher than those of the pure collagen hydrogel, indicating that its mechanical strength was superior to that of the pure collagen hydrogel material (Figure 5C), which gave it an advantage in terms of the compressive capacity in the IVD.

To examine the impact of the material on cell activity, we placed composites of CESCs and SF–collagen hydrogels in the culture medium for continuous culture. After 72 h, HE staining was performed, and microscopic observations showed that the number of rIGF1-CESC cells in the SF–collagen hydrogel was significantly greater than the number of CESC cells in SF–collagen hydrogel (Figure 5D). 5-Ethynyl-2′-deoxyuridine (EdU) staining also confirmed that rIGF1-CESC had also gained better proliferation and survival ability (Figure 5E), which enables it to continuously release a large amount of exosomes containing rIGF1, thereby promoting the proliferation of surrounding AFCs and accelerating AF repair.

### 3.6 rIGF1-CESCs@SF-collagen hydrogel attenuated IVD degeneration in rats

**FIGURE 6:**
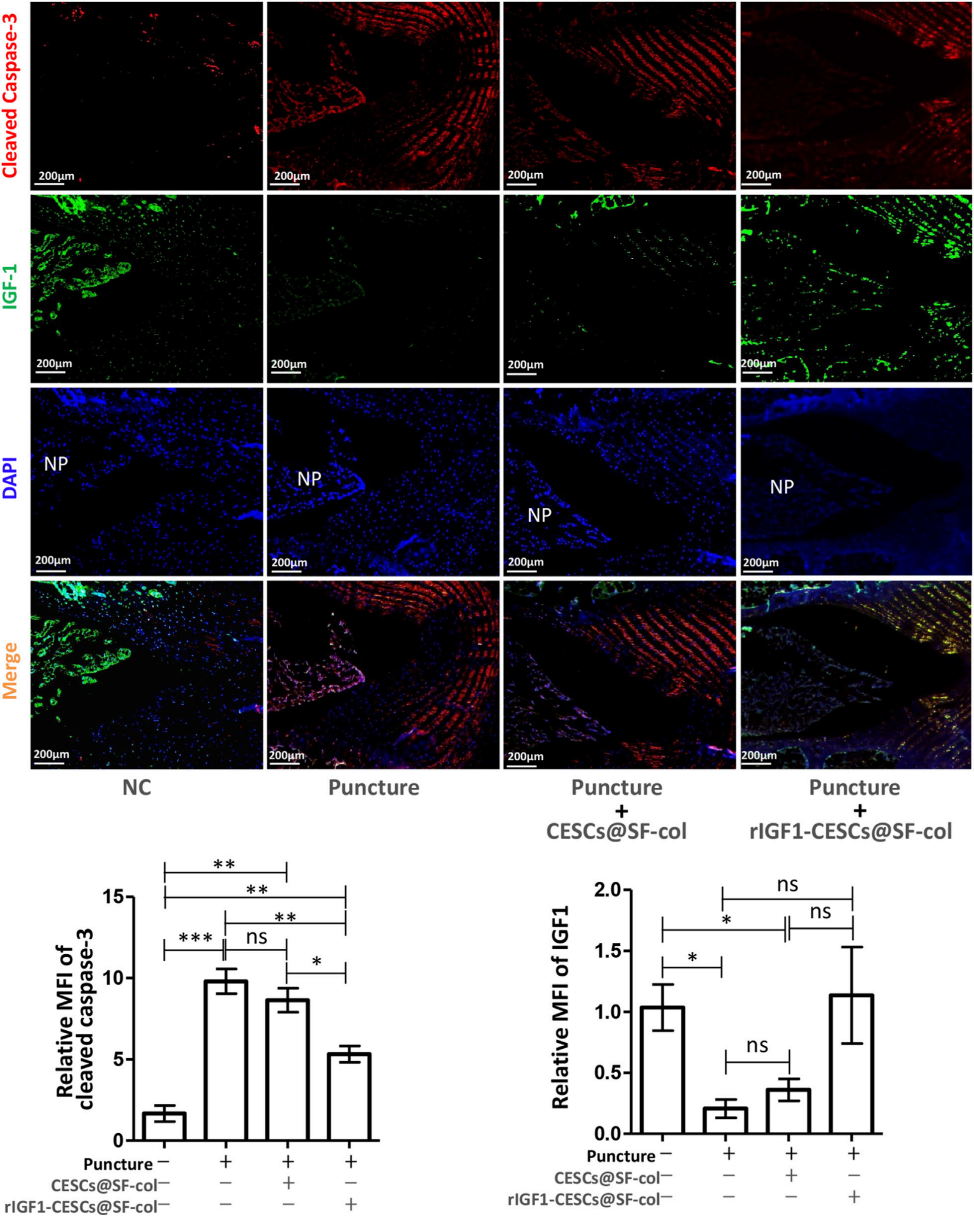
rIGF1-CESCs@SF–collagen hydrogel releases IGF1 and inhibits apoptosis of cells within IVD. Design grouping: healthy control group, puncture group, the CESCs@SF-collagen hydrogel treatment group, and rIGF1-CESCs@SF-collagen hydrogel group. A puncture damage in AF tissue and injection of hydrogel for wound treatment; 1 week later, three rats in each group were taken, the tail IVDs of rats were separated, and the cleaved caspase-3 (red) and IGF1 (green) were detected by immunofluorescence. Data are presented as the mean ± SD; *p < 0.05, **p < 0.01, and ***p < 0.001. ns: not significant by one-way ANOVA.

**FIGURE 7:**
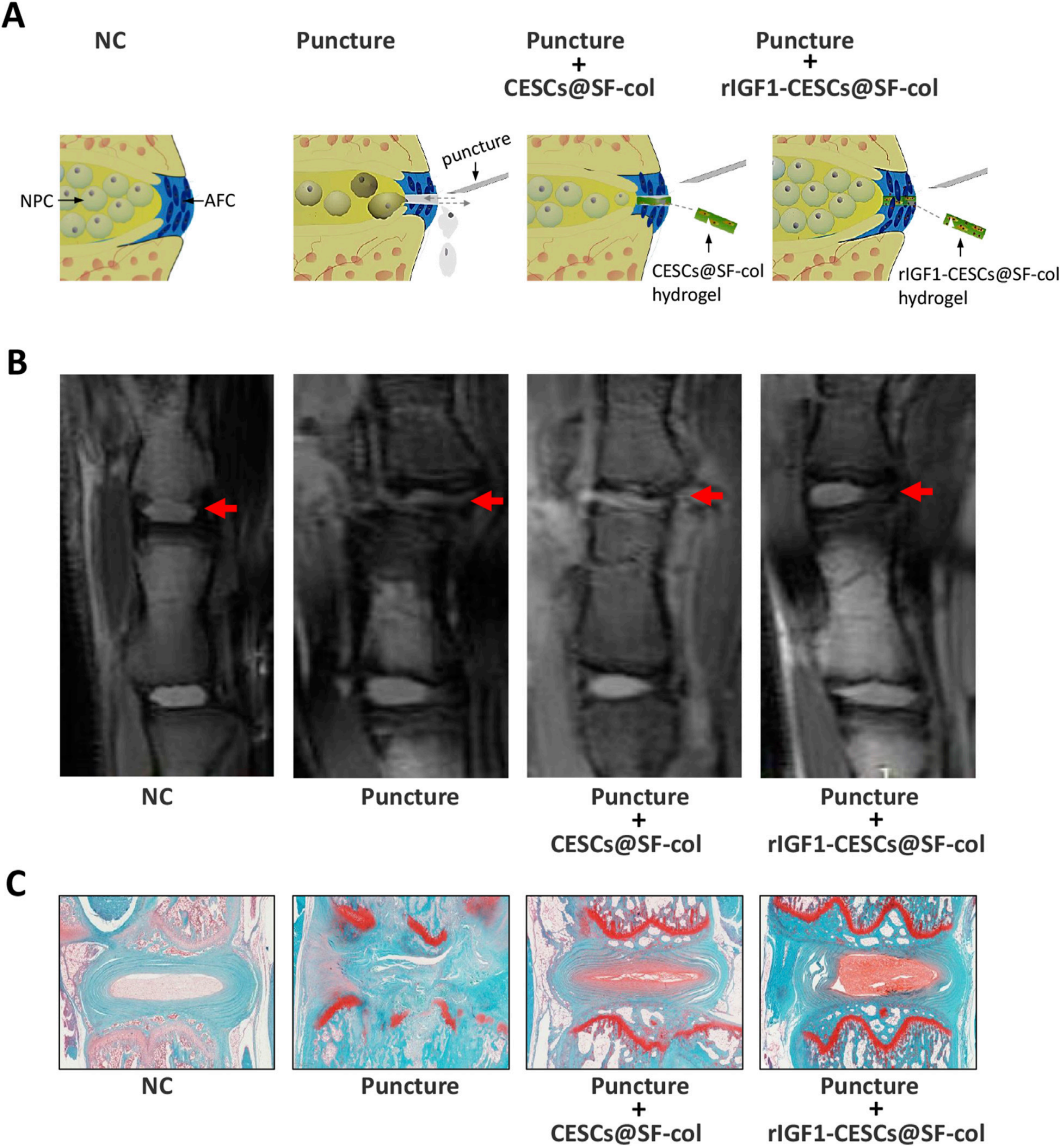
rIGF1-CESCs@SF-collagen hydrogel attenuates the degeneration of IVD in rats. **(A)** Graphic description of treatment for AF wounds with rIGF1-CESCs@SF-collagen hydrogel. **(B)** In the sixth week after AF tissues were punctured, the IVD was detected using MRI, and then they were separated. Safranin O-fast green staining **(C)**, microscopic observation, and imaging were performed. n = 3.

To verify the effects of the rIGF1–CESCs@SF–collagen hydrogel on the treatment of AF wounds, rats were divided into the following groups: the healthy control group, puncture group, the CESCs @SF-collagen hydrogel group, and the rIGF1-CESCs@SF-collagen hydrogel treatment group. A puncture was used to induce damage to the AF of the rat tail IVD. CESCs@ SF-collagen hydrogel or rIGF1-CESCs@SF-collagen hydrogel was then injected into the AF damage site. One week later, IVDs were isolated, and immunofluorescence was used to detect the protein expression and localization of cleaved caspase-3 and IGF1. After puncture, the expression of cleaved caspase-3 in the IVD tissues of each experimental group increased, and the concentration of IGF1 in the rIGF1-CESCs@SF-collagen hydrogel experimental group significantly increased (Figure 6). The elevation of IGF1 to some extent inhibits the expression of cleaved caspase-3. Six weeks later, MRI detection of tail IVD and acquisition of images (Figures 7A,B) and Safranin O-fast green staining of the isolated IVDs (Figure 7C) were carried out. The above results showed that the rIGF1–CESCs@SF–collagen hydrogel effectively repaired the damaged AF, maintained the integrity of the NP by preventing the loss and death of NPCs, maintained the height of the IVD, and significantly attenuated IVD degeneration.

## 4 Discussion

The CEP is a crucial hub connecting the IVD to the vertebrae and is an essential pathway for the nutrition and metabolism of the IVD. CESCs are derived from CEP tissues and play an important role in maintaining normal physiological functions of the entire IVD and CEP. Multiple studies have confirmed that CESCs secrete various factors (Shang et al., 2015) and extracellular vesicles (Luo et al., 2021a; Luo et al., 2021b) to regulate IVD function. In the treatment of IVD diseases using stem cells (such as mesenchymal stem cells and adipose-derived stem cells), the ECM is generated by inducing stem cell differentiation into NPCs or AFCs to induce repair in the IVD. CESCs are derived from the IVD and thus have higher compatibility than mesenchymal stem cells and adipose-derived stem cells. Based on our extensive research experience with CESCs (Luo et al., 2022; Luo et al., 2021a; Luo et al., 2021b) and SF (Luo et al., 2023; Wang et al., 2023), we performed IVD treatment experiments using these two materials.

Through proteomic mass spectrometry, we found that CESC-exosomes (CESC-Exos) were rich in rIGF1. IGF is crucial for the growth and development of bone tissues (McCarthy et al., 1989) and directly inhibits IVD degeneration (Liu et al., 2015). It has been speculated that CESCs could maintain the physiological balance of IVDs by releasing IGF1 via extracellular vesicles. Through mRNA and protein detection, we observed that IGF1 was abundantly present in mildly degenerated human CEP regions of IVD tissues, whereas it was expressed at low levels in severely degenerated tissues. The results of the rat experiments were similar to those in humans, with IGF1 being highly expressed in the CEP and NP regions of healthy rats. We isolated and cultured rat NPCs and AFCs *in vitro* and determined the effects of IGF1 on these two cell types. Because IGF1 needs to bind to the IGFR of target cells and activate multiple intracellular signaling pathways to perform its functions, we examined the expression of IGFR in these two cell types. Western blotting revealed IGFR expressions in both AF and NP tissues, with no significant differences in IGFR expression between healthy and degenerated tissues. Recombinant rIGF1 was added to AFCs and NPCs cultured *in vitro*. The MTT assay confirmed that rIGF1 effectively promoted the proliferation of AFCs and NPCs. Wound healing and transwell assays confirmed that IGF1 contributed to the migration of AFCs and NPCs. This indicates that the IGF1/IGFR pathway could activate AFCs within the IVD. Furthermore, rIGF1 was overexpressed in CESCs, and exosomes were extracted. When the latter was added to the culture medium of AFCs *in vitro*, AFC activity was enhanced. This confirms that CESCs promote the proliferation and migration of cells by activating the IGF1/IGFR signaling network of AFCs through the release of IGF1-exosomes. This provided an engineered cellular exosome that could rapidly repair damaged AF.

Furthermore, we searched for materials that could support the growth of CESCs within the IVD. SF has been widely used in studies on repair and degeneration after IVD damage (Lin et al., 2023). SF–collagen hydrogels were prepared using silk fibroin and collagen. SEM and strength tests confirmed that it had a loose and porous structure and relatively high compressive capacity, making it suitable for treating IVD tissues. EdU staining confirmed that it promotes CESC growth. To further verify the effect of the rIGF1-CESCs@SF-collagen hydrogel on the treatment of AF wounds, puncture was used to induce damage to the AF in rat IVD. Meanwhile, the rIGF1-CESCs@SF-collagen hydrogel was injected at the wound site. Immunofluorescence was used to detect the expressions of cleaved caspase-3 and IGF1. After puncture, the expression of cleaved caspase-3 in the IVD tissues of each experimental group increased, whereas the expression of IGF1 in the rIGF1-CESCs@SF-collagen hydrogel experimental group increased. The concentration of IGF1 is negatively correlated with the expression of cleaved caspase-3, and IGF1 inhibited the expression of cleaved caspase-3, suggesting that IGF1 may promote wound healing in AF tissue by inhibiting AFC cell apoptosis and promoting proliferation. MRI detection and Safranin O-fast green staining of the IVDs at 6 weeks showed that the rIGF1-CESCs@SF-collagen hydrogel significantly inhibited IVD degeneration, and it is effective for wound treatment of AF.

This indicates that the rIGF1-CESCs@SF-collagen hydrogel can accelerate the healing of AF wound tissues and prevent the loss of NP tissues. This is an ideal strategy for the treatment of IVD injury and degeneration and provides meaningful references for basic and clinical research in this field. There is a significant difference between real-world AF injury and induced injury in rat AF, which will bring unpredictable difficulties to wound treatment. However, many drawbacks of stem cells still exist, such as uncontrolled expansion and cell aging. In addition, the function of exosomes released by uncontrolled or aging stem cells is also affected, leading to uncontrollable consequences for treatment. Therefore, further exploration of CESCs to maintain the stability of their stem cells and sustain the release of functional exosomes *in vivo* is crucial (Meng et al., 2020). In addition, the immunologic responses to the activated SF–collagen hydrogel are still in the shadows. Although SF and hydrogel are generally considered medical materials with low immunogenicity, they can still induce inflammatory reaction to a certain extent, and the difference in the processing technology of SF will also have different effects on the resistance of the mammalian immune system (Majumder et al., 2024). Therefore, a large number of *in vivo* and *in vitro* experiments are still needed before the clinical application of the rIGF1-CESCs@SF-collagen hydrogel.

## Data Availability

The original contributions presented in the study are included in the article/supplementary material; further inquiries can be directed to the corresponding authors.
